# The Association Between Early Graft Function, Donor Type and Long-Term Kidney Transplant Outcomes

**DOI:** 10.3389/ti.2025.14197

**Published:** 2025-05-16

**Authors:** Karthik Venkataraman, Georgina L. Irish, Michael G. Collins, Philip A. Clayton

**Affiliations:** ^1^ Faculty of Health and Medical Sciences, The University of Adelaide, Adelaide, SA, Australia; ^2^ Central and Northern Adelaide Renal and Transplantation Service, Royal Adelaide Hospital, Adelaide, SA, Australia; ^3^ Transplant Epidemiology Group (TrEG), Australia and New Zealand Dialysis and Transplant (ANZDATA) Registry, South Australian Health and Medical Research Institute (SAHMRI), Adelaide, SA, Australia

**Keywords:** graft function, kidney transplant, ANZDATA, graft survival, delayed graft function (DGF)

## Abstract

Delayed graft function (DGF), is associated with inferior graft outcomes. Whether poor graft function without dialysis, termed slow graft function (SGF), affects outcomes is unclear. We investigated associations between SGF (serum creatinine dropping by less than 30% between days 1 and 2), DGF and graft outcomes by donor type in a cohort of 17,579 Australian and New Zealand kidney transplant recipients from 2001–2021. The primary outcomes were graft survival and death-censored graft survival Compared with immediate graft function, both SGF (Adjusted hazard ratio [aHR] 1.48 (95% CI 1.14–1.91) and DGF [aHR 1.97 (1.42–2.73)] were associated with reduced graft survival in living donor and donation after brain death (DBD) recipients [SGF aHR 1.13 (1.01–1.27); DGF aHR 1.37 (1.24–1.51)]. In donation after circulatory death (DCD) recipients, DGF [(aHR 1.52 (1.13–2.04)] but not SGF [(aHR 1.55 (1.13–2.13)] was associated with reduced graft survival. Findings were similar for death-censored graft survival. In secondary analyses, SGFwas associated with reduced patient survival in living donor recipients. SGF and DGF were associated with lower 12-month eGFR for all donor types. DGF increased the odds of rejection for all donor types; for SGF this association was significant only for DBD recipients. SGF is associated with adverse outcomes in live donor and DBD kidney recipients.

## Graphical Abstract

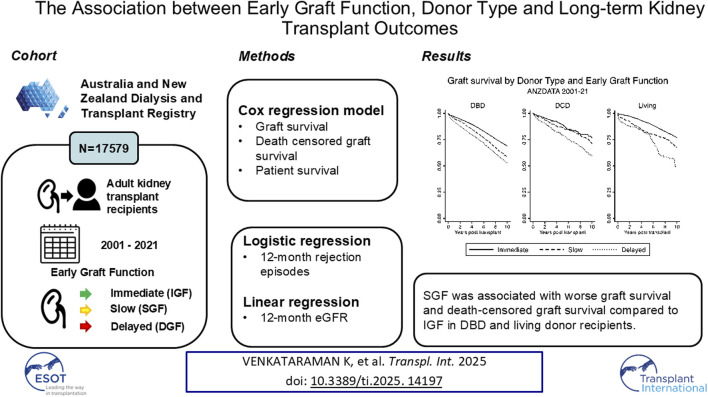

## Introduction

Kidney transplantation provides improved quality of life and improved survival, at reduced cost, when compared to dialysis as a kidney failure treatment [1–3]. The function of the transplant graft in the days after kidney transplantation, termed early graft function, has important clinical implications. Poor EGF is associated with increased post-transplant dialysis sessions, increased days in hospital and increased resource utilisation [4, 5]. In addition, poor early graft function may influence clinical decision-making around calcineurin inhibitor dosing and result in interventions such as kidney biopsy, thus exposing patients to the complications associated with these interventions [6, 7].

Early graft function can be broadly categorised into immediate graft function (IGF), slow graft function (SGF) or delayed graft function (DGF) [8, 9]. DGF is widely defined as the requirement for dialysis within 1 week of transplantation [10–12]. SGF is characterised by slower-to-improve graft function, when compared to IGF, without the need for dialysis. In essence, SGF can be thought to exist on a spectrum between IGF and DGF [13]. In this, SGF is similar to the concept of functional DGF (fDGF) described in the literature [14, 15]. Both SGF and fDGF have had varying definitions in the literature.

DGF is linked to poorer graft survival and increased episodes of early rejection [10, 16, 17]. SGF has also been linked to poorer graft outcomes in some studies [7, 18–20] but not in others [21, 22] resulting in uncertainty regarding its clinical significance. This may be linked to the aforementioned variability in definition [21, 23, 24]. Outcomes after SGF may vary by donor type, with some studies showing it portends a poorer prognosis in LD transplants [25–27]. However, previous studies have been underpowered to assess the effect of donor type on the association between SGF and long term graft outcomes. Additionally, there is uncertainty on the magnitude of effect that DGF and SGF have on long-term patient survival [7, 10].

We hypothesized that, compared to IGF, both SGF and DGF are associated with reduced long term graft survival and death censored graft survival in recipients of a kidney transplant.

## Patients and Methods

### Study Population

We included all adult (aged ≥18 years) recipients of kidney-alone transplants performed in Australia and New Zealand between 2001 and 2021 from the Australia and New Zealand Dialysis and Transplant (ANZDATA) Registry. Transplants that occurred outside Australia and New Zealand, pathological donors (defined as kidneys transplanted after nephrectomy for tumour), patients that experienced primary graft failure (i.e., graft loss within 7 days) and multi-organ transplants were excluded.

### Early Graft Function Definitions

The definitions of DGF and SGF used were as recorded by the ANZDATA registry. Prior to 2017, SGF was defined as the absence of a spontaneous fall in serum creatinine of >10% within 72 h of transplant, without the need for dialysis; DGF was the requirement for dialysis within 72 h post-transplantation. IGF in this era was defined as a spontaneous fall in serum creatinine by over 10% within 72 h of transplantation. From 2017 onwards, these definitions were updated to align with internationally accepted definitions: DGF was defined as requirement for dialysis within 7 days of transplant, and SGF by a reduction in serum creatinine of ≤30% between day one and day two post transplantation. IGF in this era was defined as a spontaneous fall in serum creatinine by over 30% by day 2 post transplantation. We included an adjustment for transplantation era in our statistical analysis models to account for the change in definitions, and also assessed for interactions between era and early graft function in the different models.

### Clinical Outcomes

The primary outcomes assessed were; a) graft survival, defined as time from transplantation until return to dialysis, repeat kidney transplantation or death with a functioning graft and b) death-censored graft survival (DCGS), defined as time from transplantation until return to dialysis or repeat kidney transplantation, censored for death with a functioning graft.

The secondary outcomes assessed were; a) patient survival, defined as the time from date of transplantation to patient death and not censored at graft failure, b) 12 months estimated glomerular filtration rate (eGFR), calculated using the original CKD Epidemiology Collaboration (CKD Epi) equation [28] and c) acute rejection at 12 months, defined as any episode of acute rejection (either biopsy proven or suspected) at 12 months respectively, as reported to the registry [16].

### Data Variables

Baseline recipient characteristics obtained from the ANZDATA registry included age, gender, ethnicity, primary kidney disease, body mass index (BMI), time on dialysis, repeat transplantation, calculated panel reactive antibodies and comorbid conditions (smoking status, diabetes mellitus, ischaemic heart disease, peripheral vascular disease, cerebrovascular disease and chronic lung disease).

Baseline donor characteristics obtained from the ANZDATA and the Australia and New Zealand Organ Donor (ANZOD) and Australian and New Zealand Living Kidney Donor registries included age, sex, BMI and comorbid conditions (smoking status, hypertension and diabetes mellitus). Donors were classified as either living donor (LD), donation after brain death (DBD) or donation after circulatory death (DCD).

Transplant related characteristics obtained included total ischaemic time, ABO compatibility status and number of human leukocyte antigen (HLA) mismatches at the A, B and DR loci.

All comorbidities were from the ANZATA survey the year prior to transplantation.

### Statistical Analysis

Continuous variables were reported as mean and standard deviation, or median and interquartile range, as appropriate. Categorical variables were reported as counts and percentages. We created Kaplan Meier Curves for all survival outcomes. We hypothesised that the association between early graft function would differ by different donor types. To account for this difference, *a priori* strata were assumed between donor type and early graft function (i.e., the baseline hazard will be constant only within the donor types). We used stratified Cox proportional hazard models for all survival outcomes. All survival times were censored at the end of follow-up on 31 December 2021. All variables were assessed for linearity through categorisation of continuous variables and Martingale Residuals. For graft survival and death censored graft survival, age was non-linear and transformed using fractional polynomials. We hypothesised that due to a change in how ANZDATA collected SGF over time there may be a difference in the association between early graft function and the different outcomes by era. To investigate this, we assessed for an interactions between early graft function subtype and era (years 2001–2016 vs. years 2017–2021) using forward elimination with a threshold p value of 0.1 (Supplementary Figure S3). The donor variables assessed for inclusion in the models were age, sex, BMI, hypertension, smoking, and diabetes mellitus. The recipient variables assessed for inclusion in the models were age at transplant, recipient sex, graft number, years on dialysis, ischaemic time, peak PRA, primary kidney disease, BMI, smoking, peripheral vascular disease, diabetes mellitus, ischaemic heart disease, cerebrovascular disease, chronic lung disease and number of HLA mismatches. No interactions were found. Non-significant variables were excluded from the model using backward elimination with a threshold p value of 0.157 [29]. The proportional hazard assumption was assessed using Schoenfeld residuals. During the creation of the models, implausible values for included variables, including donor body mass index (BMI) > 80 or <10 (8), ischaemic time >40 h (4), recipient BMI >50 kg/m^2^ (11) and height under 100 cm (48) were considered missing. There were 71 (<0.5%) such implausible values that were considered missing. Given the low rate of missingness we did not perform additional analyses accounting for missingness using multiple imputation.

A fixed effects linear regression model, with fixed effects for donor type was created for the outcome of 12-month eGFR. Collinearity was assessed using the variance inflation factor. The linearity assumption for continuous variables was assessed using scatter plots of residual values.

A fixed effect logistic regression model, with fixed effects for donor type was created for the outcome of 12-month rejection. Collinearity was assessed with the variance inflation factor. The linearity assumption was assessed using categorisation of all continuous variables for covariates. For both logistic and linear regression models, interactions were assessed for using the forward elimination method. Backward elimination was used to remove non-significant variables with a threshold p value of 0.157 [29]. All models are available in the Supplementary Material. The analyses were conducted in Stata/IC 17.0 (Stata Corp, College Station TX).

## Results

### Study Population

**FIGURE 1:**
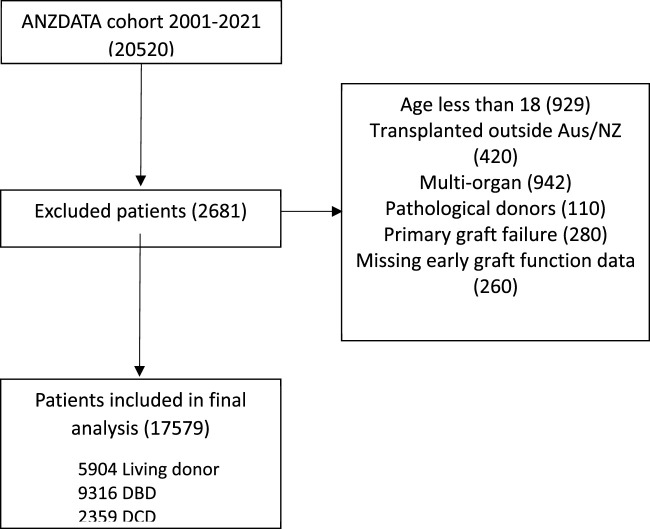
Cohort diagram (DBD, donation after brain death; DCD, donation after circulatory death).

Between January 2001 and December 2021, a total of 20,520 transplants were performed in Australia and New Zealand and reported to the ANZDATA registry (Figure 1). 2941 transplant recipients were excluded: 260 recipients with missing early graft function data, 929 recipients aged <18 years, 420 transplants which occurred outside of Australia and New Zealand, 942 multi-organ transplants, 110 pathological donors, and 280 primary graft failures. A total of 17,579 transplants were included in this study, comprised of 2,359 (13.4%) donation after circulatory death (DCD) transplants, 9,316 (53.0%) donation after brain death (DBD) transplants and 5,904 (33.6%) living donor transplants. The baseline characteristics of the study population are described in Table 1. The median follow-up time was 6.8 (IQR 3.3–11.6) years. The proportion of recipients with DGF was 3,604/17,579 (20.5%), the proportion with SGF was 2277/17,579 (12.9%), and the proportion with IGF was 11,698/17,579 (66.6%). During the follow up period, there were 2,434 (13.9%) deaths and 2,575 (14.7%) experienced graft loss. 243 (1.38%) recipients were lost to follow up.

**TABLE 1:** Patient characteristics (IGF, Immediate Graft Function; SGF, Slow Graft Function, Delayed Graft Function; DCD, Donation after circulatory death; DBD, Donation after brain death; LD, living donor).

Characteristic	IGF	SGF	DGF	p-value
N	11,698	2,277	3,604	
Age at transplant, median (IQR)	49 (38, 59)	53 (43, 61)	54 (44, 62)	<0.001
Recipient Male	7,118 (60.8%)	1,518 (66.7%)	2,451 (68.0%)	<0.001
Recipient Ethnicity				<0.001
Unknown	584 (5.0%)	111 (4.9%)	185 (5.1%)	
White/European	8,513 (72.8%)	1,600 (70.3%)	2,381 (66.1%)	
Aboriginal/Torres Strait Islander	280 (2.4%)	92 (4.0%)	218 (6.0%)	
Maori	334 (2.9%)	67 (2.9%)	108 (3.0%)	
Pacific	365 (3.1%)	86 (3.8%)	150 (4.2%)	
Asian	1,342 (11.5%)	257 (11.3%)	441 (12.2%)	
Other	280 (2.4%)	64 (2.8%)	121 (3.4%)	
Primary Renal Disease				<0.001
GN	5,190 (44.8%)	917 (40.6%)	1,440 (40.1%)	
Polycystic	1,665 (14.4%)	321 (14.2%)	429 (12.0%)	
Reflux	1,036 (8.9%)	165 (7.3%)	251 (7.0%)	
Hypertension	674 (5.8%)	147 (6.5%)	248 (6.9%)	
Diabetes	1,223 (10.6%)	339 (15.0%)	665 (18.5%)	
Other	1790 (15.5%)	369 (16.3%)	556 (15.5%)	
Recipient Smoker	4,566 (39.8%)	1,028 (45.8%)	1,662 (46.8%)	<0.001
Recipient Diabetes Meillitus	1828 (15.7%)	497 (21.9%)	994 (27.6%)	<0.001
Recipient Ischaemic heart disease	1,680 (14.4%)	488 (21.5%)	877 (24.4%)	<0.001
Recipient Peripheral vascular disease	840 (7.2%)	231 (10.2%)	486 (13.5%)	<0.001
Recipient Cerebrovascular disease	566 (4.8%)	152 (6.7%)	248 (6.9%)	<0.001
Recipient Chronic lung disease	772 (6.6%)	217 (9.6%)	384 (10.7%)	<0.001
Recipient Body Mass Index (BMI) kg/m^2^, median (IQR)	25.9 (22.8, 29.4)	27.1 (24.0, 30.5)	27.8 (24.4, 31.5)	<0.001
Time on dialysis (years), median (IQR)	1.6 (0.5, 3.5)	2.6 (1.2, 4.8)	3.4 (2.0, 5.4)	<0.001
Total ischaemia (to nearest hour), median (IQR)	6 (3, 12)	11 (7, 15)	12 (8, 15)	<0.001
ABO incompatible transplant	552 (4.7%)	31 (1.4%)	33 (0.9%)	<0.001
Graft number >1	1,337 (11.4%)	261 (11.5%)	533 (14.8%)	<0.001
HLA-A mismatch				<0.001
0	2,615 (22.7%)	437 (19.3%)	633 (17.6%)	
1	5,680 (49.2%)	1,098 (48.4%)	1,627 (45.2%)	
2	3,248 (28.1%)	734 (32.3%)	1,339 (37.2%)	
HLA-B mismatch				<0.001
0	1819 (15.8%)	320 (14.1%)	469 (13.0%)	
1	5,176 (44.8%)	890 (39.2%)	1,263 (35.1%)	
2	4,547 (39.4%)	1,059 (46.7%)	1867 (51.9%)	
HLA-DR mismatch				<0.001
0	3,452 (30.0%)	707 (31.2%)	1,008 (28.0%)	
1	4,972 (43.1%)	843 (37.3%)	1,301 (36.2%)	
2	3,101 (26.9%)	713 (31.5%)	1,286 (35.8%)	
Any induction therapy	9,541 (%)	1965 (%)	3,139 (%)	<0.001
Donor type				<0.001
DCD	543 (23.0%)	518 (22.0%)	1,298 (55.0%)	
DBD	5,672 (60.9%)	1,493 (16.0%)	2,151 (23.1%)	
Living	5,483 (92.9%)	266 (4.5%)	155 (2.6%)	
Paired kidney Exchange	400 (89.3%)	27 (6.0%)	21 (4.7%)	
Donor age, median (IQR)	48 (36, 57)	52 (40, 61)	51 (40, 60)	<0.001
Donor Male	5,481 (49.2%)	1,206 (53.5%)	2,118 (59.1%)	<0.001
Donor Body Mass Index (BMI) kg/m2, median (IQR)	26.1 (23.6, 29.3)	26.7 (24.0, 30.0)	27.4 (24.4, 31.1)	<0.001
Donor Smoker	5,597 (51.0%)	1,353 (60.3%)	2,245 (62.7%)	<0.001
Donor Hypertension	1,650 (15.1%)	581 (26.1%)	1,022 (28.9%)	<0.001
Donor Diabetes Meillitus	283 (2.6%)	118 (5.3%)	238 (6.7%)	<0.001

### Primary Endpoints

**FIGURE 2:**
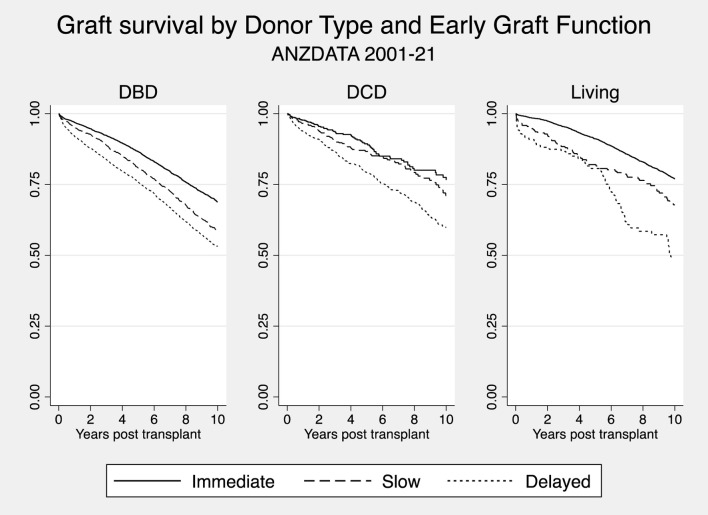
Kaplan Meier Graft Survival curves by donor type, stratified by early graft function subtypes (DCD, Donation after circulatory death; DBD, Donation after brain death; Living, living donor.

Figure 2 shows the Kaplan-Meier curves, comparing graft survival by donor type, stratified by early graft function subtype. DGF and SGF in living donor recipients and recipients of DBD transplants, but not DCD transplants, were associated with a reduction in graft survival when compared to recipients with immediate graft function. Table 2 shows the multivariable analyses of the primary endpoints, along with patient survival, stratified by donor type.

**TABLE 2:** Adjusted associations between SGF and DGF and Graft Survival, Death Censored Graft Survival (DCGS) and Patient Survival (SGF, slow graft function; DGF, delayed graft function; DBD, donation after brain death; DCD, donation after circulatory death; aHR, adjusted hazard radio.

	Graft Survival			DCGS			Patient surivival		
	aHR	95% CI	p value	aHR	95% CI	p value	aHR	95% CI	p value
LD									
SGF	1.47	1.14, 1.91	<0.001	1.53	1.11, 2.11	0.011	1.55	1.13, 2.13	0.007
DGF	1.97	1.42, 2.73	<0.001	1.93	1.27, 2.94	0.003	2.01	1.37, 2.94	<0.001
DBD									
SGF	1.13	1.01, 1.27	0.008	1.33	1.15, 1.55	<0.001	1.02	0.90, 1.16	0.694
DGF	1.37	1.24, 1.51	<0.001	1.49	1.31, 1.70	<0.001	1.29	1.16, 1.44	<0.001
DCD									
SGF	1.01	0.70, 1.44	0.596	1.23	0.74, 2.03	0.44	1.03	0.69, 1.53	0.869
DGF	1.52	1.14, 2.04	<0.001	1.80	1.19, 2.73	0.006	1.47	1.06, 2.04	0.017

SGF [aHR 1.48 (95% CI 1.14, 1.91)] and DGF [aHR 1.97 (95% CI 1.42, 2.73)] were associated with increased graft loss in living donors (Supplementary Figure S4). Both SGF [aHR 1.13 (95% CI 1.01, 1.27)] and DGF [aHR 1.37 (95% CI 1.24, 1.51)] were associated with increased graft loss when compared to IGF in DBD transplant recipients. In DCD transplant recipients, DGF [aHR 1.52 (95% CI 1.13, 2.04)] was associated with increased graft loss. However, in DCD transplant recipients, there was no statistically significant difference between SGF [aHR 1.01 (95% CI 0.70, 1.44)] and IGF.

Similarly, when assessing death censored graft loss (Supplementary Figure S5), SGF [aHR 1.53 (95% CI 1.11, 2.11)] and DGF [aHR 1.93 (95% CI 1.27, 2.95)] were associated with increased death censored graft loss in living donors. Both SGF [aHR 1.33 (95% CI 1.15, 1.55)] and DGF [aHR 1.49 (95% CI 1.31, 1.70)] were associated with increased death censored graft loss when compared to IGF in DBD transplant recipients. In DCD transplants, DGF [aHR 1.80 (95% CI 1.19, 2.73)] was associated with increased death censored graft loss. SGF [aHR 1.23 (95% CI 0.74, 2.03)] was not significantly associated with death censored graft loss in DCD transplants.

### Secondary Endpoints

#### Patient Survival

Both DGF [aHR 2.01 (95% CI 1.37, 2.94)] and SGF [aHR 1.55 (95% CI 1.13, 2.13)] were associated with decreased patient survival in living donor transplant recipients (Supplementary Figure S6). DGF was associated with decreased patient survival in both DBD [aHR 1.29 (95% CI 1.17, 1.44)] and DCD [aHR 1.47 (95% CI 1.06, 2.04)] transplant recipients. SGF was not associated with decreased survival in DBD [aHR 1.02 (95% CI 0.90, 1.16)] or DCD [aHR 1.03 (95% CI 0.69, 1.53)] transplant recipients.

#### Graft Function

**FIGURE 3:**
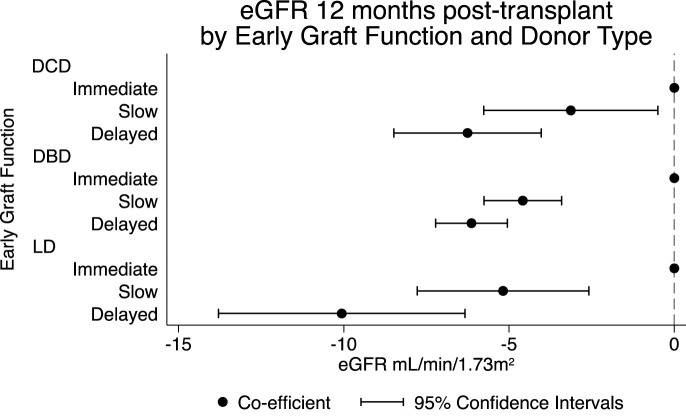
Association between early graft function stratified by donor type donor type on eGFR at 12-month post-transplant adjusted model.

For all donor types, SGF and DGF were associated with lower eGFR at 12-month post-transplant (Figure 3). In living donors, SGF was associated a reduction in eGFR at 12 months of 5.2 mL/min (95% CI 2.6–7.8) and DGF was associated with a reduction in eGFR at 12 months of 10.1 mL/min (95% CI 6.3–13.8). In DBD recipients, SGF was associated a reduction in eGFR at 12 months of 4.6 mL/min (95% CI 3.4–5.8) and DGF was associated with a reduction in eGFR at 12 months of 6.1 mL/min (95% CI 5.1–7.2). In DCD recipients, SGF was associated a reduction in eGFR at 12 months of 3.1 mL/min (95% CI 0.5–5.8) and DGF was associated with a reduction in eGFR at 12 months of 6.3 mL/min (95% CI 4.0–8.5).

#### Rejection

**FIGURE 4:**
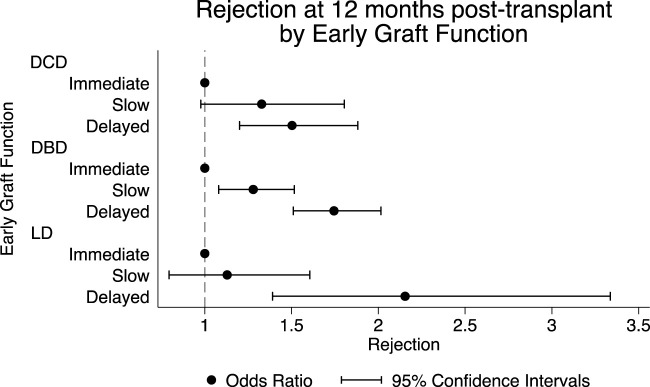
Episodes of rejection at 12 months, stratified by donor type and early graft function adjusted model.

Figure 4 shows the association between early graft function and episodes of rejection at 12 months. In DBD recipients, both SGF [OR 1.28 (95% CI 1.08–1.52) and DGF [OR 1.74 (95% CI 1.51–2.02) were associated with an increased odds of rejection at 12 months. For DCD recipients, DGF was associated with increased odds of rejection at 12 months [OR 1.50 (95% CI 1.20–1.88)]. However, SGF was not associated with a statistically significant increase in the odds of rejection [OR 1.32 (95% CI 0.98–1.80)]. In recipients of living donors, DGF was associated with an increased odds of rejection [OR 2.15 (95% CI 1.39– 3.34). However, SGF was not significantly associated with rejection at 12 months [OR 1.13 (95% CI 0.79–1.61)].

## Discussion

In this study, involving 17,579 kidney transplant recipients, we showed that both DGF and SGF are associated with poorer graft outcomes after kidney transplantation. SGF is associated with worse graft survival and death censored graft survival in living and DBD, but not DCD, recipients. This study also demonstrates that SGF is associated with worse patient survival in live donor recipients when compared to IGF. Additionally, we demonstrated that both SGF and DGF are associated with increased risk of early rejection and worse eGFR at 12 months post transplantation.

Importantly, this study demonstrates that the associations between early graft function and long-term outcomes differ by different donor types, and shows evidence for the consequences of SGF in DCD, DBD and living donor kidney transplants. In the DBD cohort, SGF was associated with reduced graft survival and increased rejection, but was not associated with worse patient survival. This is consistent with previous findings in deceased donor transplantation [7, 20, 30]. We did not find an association between SGF and adverse graft survival in DCD transplants. The point estimate of hazard ratio for DCGS (1.22) does not exclude an adverse association that this study was underpowered to find. Additionally, the reduction in 12-month eGFR and increased episodes of rejection suggest some clinically meaningful associations of SGF in DCD transplants.

The association of SGF with poor long term graft outcomes in living donors is consistent with findings in smaller, singe-center studies [25, 26, 31]. Our study confirms and expands on this prior literature using data from a large multi-centre registry analysis. Kinoshita et. al. assessed 10-year graft survival in 272 living donor transplant recipients with and without SGF, defined as a CRR on day 2 of less than 30% [25]. They reported decreased graft survival at 5 and 10 years, however, did not find a difference in rejection rates or eGFR at 12 months or a statistically significant change in eGFR at 12 months. We also did not find an increased odds of rejection in living donor recipients with SGF when compared to living donor recipients with IGF, but found a decrease in eGFR at 12 months. Lee et al. reported 10-year graft outcomes in 310 living donor transplants and found that the decreased graft survival seen in living donor transplant recipients with SGF appeared to be associated with an increased incidence of acute rejection [32]. In contrast, our findings suggest that the reduction in graft survival is not mediated through rejection, as we did not find an increase in odds of rejection in living donors with SGF but did find significant reduction in graft survival.

Our finding of a reduction in patient survival in living donors with SGF has not been reported previously. Live donor surgery is undertaken in a planned, elective fashion, and typically involves a short cold ischaemic time. As such, perioperative and recipient factors may be more significant factors in the development of SGF and DGF in these transplants in comparison to the deceased donor setting, where donor characteristics and organ storage play a highly significant role. This may allude to perioperative morbidity impacting both early graft function and patient survival. It is also possible that there are other yet to be identified factors that play a role. Further studies in different populations and settings should be performed to confirm and validate our findings.

Consistent with the findings of prior studies using ANZDATA, and conducted in other countries and settings, we found that DGF is associated with poor graft outcomes and reduced patient survival across donor types [10, 17, 33, 34]. While the adverse consequences of DGF have been well established, the consequences of SGF remain less clearly elucidated. The binary nature of the accepted definition of DGF, which is defined by the requirement for dialysis within the first week in most studies, makes DGF an easily identifiable entity in clinical settings [35]. SGF, which is characterised clinically by poor kidney function measured biochemically without the need for dialysis, has been more variably defined in the literature [8]. The heterogeneity of reported outcomes associated with SGF may reflect the heterogeneity in definitions of SGF. A study by Hall et. al. correlated the various definitions of SGF in the literature and found that a creatinine over 2.5 mg/dL (221 μmol/L) at day 7 post-transplant or a creatinine reduction ratio (CRR) between days 1 and 2 of <25% had the best correlation to the eGFR at 12 months [8]. While eGFR at 12 months is a surrogate endpoint and definitions of SGF have not been validated against harder clinical endpoints such as graft survival, this study provides support in the use of the CRR between days 1 and 2 by 30% as a definition for SGF. It is the nature of these definitions of early graft function to take the continuum of graft function between IGF and DGF and create categorically definable entities. While these distinctions are artificial, as long as they represent clinically distinct phenotypes, these definitions are important.

Previous studies suggesting a link between SGF and long-term graft outcomes have largely consisted of single centre, or small multi-centre observational studies that each included fewer than 1,500 patients [9, 18–20, 22, 30, 31, 36–38]. While several of these studies have shown an association between SGF and graft survival at 5–10 years or eGFR at 12 months, most have been underpowered to evaluate these associations in subgroups of donor types. The results of our study support the findings from existing larger cohort studies. Wang et al assessed the association between SGF and long-term graft survival and death censored survival, as well as all-cause mortality in 1,222 recipients of both living and deceased donor kidney transplants, using two different definitions of SGF. This study suggested that both definitions of SGF were associated with worse graft survival and DCGS, but not worse mortality.

Our findings demonstrate that SGF has important implications for clinical practice. Simply dichotomising early graft function into DGF or IGF is an oversimplification, which results in inattention to the clinically significant adverse effects of SGF. Recognising SGF as a distinct clinical entity with associated poor outcomes is an important step towards improving long term graft outcomes. Recent interventions have been shown to reduced DGF, such as balanced crystalloids [39] and machine perfusion [40]. Similarly, there may be interventions that reduce SGF. The magnitude of impact of SGF appears to vary across donor type, with the data demonstrating that SGF appears particularly significant in living donor transplant recipients.

In addition to these clinical implications, this evidence for the importance of SGF has important implications for clinical research. SGF may be an important intermediate end point that has the potential to be used in clinical trials, in addition to DGF, as a surrogate for long-term graft outcomes. Future work is needed to assess the impact of interventions that reduce rates of SGF on long term graft outcomes.

Our study has several strengths. It includes data on DGF and SGF from the largest cohort of transplant recipients to date and provides robust evidence for the association between early graft function and long-term graft outcomes. This study reports key patient-centred outcomes including survival and graft loss, as well as frequently reported surrogate measures such as 12-month graft function [41, 42]. The results increase the certainty of evidence for the observation that SGF represents a clinically significant intermediate phenotype between immediate graft function and DGF. Our findings also highlights the implications that SGF has for different donor types, with increasing clinical relevance in the DBD and living donor transplant recipient cohorts, compared to DCD transplant recipients. This is also the first such study that has been adequately powered to detect clinical differences in outcomes between donor types.

Several limitations must be noted. The data are retrospective and observational, and thus there is the potential for residual confounding. As a registry study, it is reliant on accurate data capture, and there is evidence that registry recorded data on co-morbidities vary from those recorded in hospital administrative datasets [43]. However, despite this, the predictive power of registry-recorded co-morbidity data for mortality and other outcomes has been demonstrated to be robust [43]. The definition of SGF recorded in the ANZDATA registry was changed in 2017, and this may have affected our analysis. While our modelling controlled for the effects of transplantation era, this change in definition might have resulted in some misclassification.

In conclusion, both SGF and DGF represent meaningful clinical entities with significant implications for patient outcomes. SGF is associated with poorer long-term graft outcomes in DBD and living donor kidney transplant recipients, as well as reduced patient survival in living donor recipients. Further research is needed to assess if interventions that improve early graft function and avoid SGF could lead to better graft survival, improved patient survival in recipients of living donor transplants, and better healthcare resource utilisation.

## Data Availability

Data may be available on request, subject to ANZDATA policies. Request to be made to corresponding author.

## Abbreviations

ANZDATA, Australia and New Zealand Dialysis and Transplant Registry; BMI, body mass index; DBD, donation after brain death; DCD, donation after circulatory death; DGF, delayed graft function; eGFR, estimated glomerular filtration rate; IGF, immediate graft function; LD, living donor; SGF, slow graft function.
