# Supplementary material for: The Association Between Early Graft Function, Donor Type and Long-Term Kidney Transplant Outcomes
Source: Transpl Int. 2025 May 16;38:14197. doi: 10.3389/ti.2025.14197 (PMC12122319; doi:10.3389/ti.2025.14197)
Supplement: Supplementary file 1 [file DataSheet1.docx]

Capsule sentence

Slow graft function (SGF) is when a kidney transplant doesn’t work as well as expected, but dialysis is not required. The long-term effects of SGF have been unclear. This study shows that SGF is associated with adverse long term outcomes.

Supplementary Figures and Tables

Supplementary Figure 1: Patient survival by EGF and donor type

Supplementary Figure 2 :DCGS by EGF and donor type


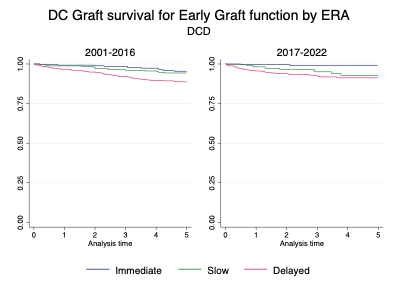

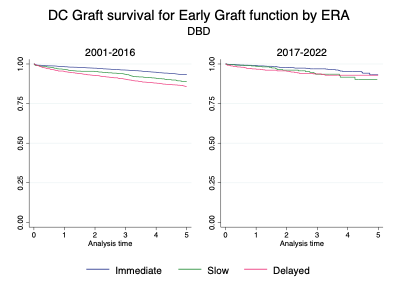

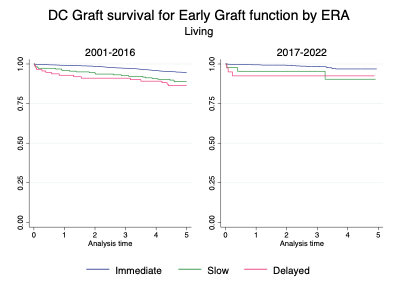


c)

b)

a)

Supplementary Figure 3: Kaplan Meier Graphs for Death Censored Graft Function stratified by era of SGF definition a) DCD, b)DBD and c)LD

Supplementary Figure 4:Adjusted Hazard ratios for graft loss by donor type and early graft function (DCD – Donation after circulatory death, DBD – Donation after brain death, LD – living donor, aHR adjusted Hazard ratios)

Supplementary Figure 5:adjusted Hazard ratios for death censored graft loss by donor type and early graft function (DCD – Donation after circulatory death, DBD – Donation after brain death, LD – living dono, aHR :adjusted Hazard ratios r)

Supplementary Figure 6: aHazard ratios for patient survival by donor type and early graft function (DCD – Donation after circulatory death, DBD – Donation after brain death, LD – living donor)

| Model | Variables included |
| --- | --- |
| Graft survival | Donor age, donor hypertension, graft number, years on dialysis, ischaemic time, panel reactive antigens, HLA DR mismatches, transplantation era ( 2017 and prior, after 2017), recipient age at transplant, primary renal disease, recipient hypertension, recipient diabetes, recipient smoking, recipient peripheral vascular disease, recipient ischaemic heart disease, recipient cerebrovascular disease. |
| Death censored graft survival | Donor age, donor gender, donor smoking, graft number, years on dialysis, panel reactive antigens, HLA mismatches, transplantation era ( 2017 and prior, after 2017), recipient age at transplant, primary renal disease, recipient hypertension, recipient diabetes, recipient smoking, recipient peripheral vascular disease, recipient BMI. |
| Patient Survival | Donor age, donor hypertension, donor smoking, graft number, years on dialysis, ischaemic time, panel reactive antigens, HLA DR mismatches, transplantation era ( 2017 and prior, after 2017), recipient age at transplant, primary renal disease, recipient hypertension, recipient gender, recipient diabetes, recipient smoking, recipient peripheral vascular disease, recipient ischaemic heart disease, recipient cerebrovascular disease, recipient chronic lung disease |
| eGFR at 12 month | Donor age, donor hypertension, donor gender, donor BMI, donor smoking, donor eGFR, graft number, years on dialysis, ischaemic time, panel reactive antigens, recipient age at transplant, primary renal disease, recipient hypertension, recipient diabetes, recipient smoking, recipient ischaemic heart disease, recipient cerebrovascular disease. |
| Rejection at 12 months | Donor age, donor gender, donor BMI, graft number, years on dialysis, ischaemic time, panel reactive antigens, HLA mismatches, recipient age at transplant, primary renal disease, recipient gender, recipient BMI, recipient smoking, induction agent |

Supplementary Table 1: Variable included in the creation of models (HLA: Human Leukocyte Antigen, BMI: Body Mass Index)

| **Parameter** | **Co-efficient** | **Lower 95% CI** | **Upper 95% CI** | **p value** |
| --- | --- | --- | --- | --- |
| Donor Age | 0.02 | 0.02 | 0.02 | <0.001 |
| Donor hypertension | 0.09 | 0.00 | 0.18 | 0.04 |
| Donor sex | -0.09 | -0.18 | 0.00 | 0.05 |
| Graft number > 1 | 0.20 | 0.04 | 0.35 | 0.01 |
| Years on dialysis | 0.03 | 0.02 | 0.05 | <0.001 |
| Panel Reactive Antibodies | 0.01 | 0.00 | 0.01 | <0.001 |
| Recipient age (fractional polynomial) | -0.73 | -0.96 | -0.50 | <0.001 |
| Recipient age (fractional polynomial) | 0.04 | 0.02 | 0.07 | <0.001 |
| Primary renal disease |  |  |  |  |
| Glomerular disease | 0.06 | -0.04 | 0.16 | 0.26 |
| Reflux | 0.00 | -0.15 | 0.14 | 0.95 |
| Hypertension | 0.14 | -0.01 | 0.29 | 0.07 |
| Diabetes | 0.26 | 0.09 | 0.42 | <0.001 |
| Other | 0.20 | 0.08 | 0.32 | <0.001 |
| Recipient Smoker | 0.29 | 0.23 | 0.35 | <0.001 |
| Recipient Peripheral vascular disease | 0.24 | 0.14 | 0.34 | <0.001 |
| Recipient Diabetes mellitus | 0.30 | 0.17 | 0.42 | <0.001 |
| Recipient Ischaemic heart disease | 0.17 | 0.08 | 0.25 | <0.001 |
| Recipient Cerebrovascular disease | 0.13 | 0.01 | 0.25 | 0.03 |
| Recipient Chronic liver disease | 0.18 | 0.07 | 0.28 | <0.001 |
| 1 HLA-DR mismatch | 0.16 | 0.08 | 0.23 | <0.001 |
| 2 HLA-DR mismatches | 0.19 | 0.11 | 0.27 | <0.001 |
| Era of transplantation | -0.26 | -0.39 | -0.12 | <0.001 |
| Interaction terms |  |  |  |  |
| DCD Slow graft function | 0.09 | -0.24 | 0.43 | 0.59 |
| DCD Delayed Graft function | 0.52 | 0.25 | 0.79 | <0.001 |
| DBD Slow graft function | 0.14 | 0.03 | 0.24 | 0.01 |
| DBD Delayed Graft function | 0.33 | 0.24 | 0.41 | <0.001 |
| Living Slow graft function | 0.44 | 0.20 | 0.68 | <0.001 |
| Living Delayed Graft function | 0.74 | 0.44 | 1.04 | <0.001 |

Graft survival by Early Graft Function

Supplementary Table 2:Coefficients, 95% confidence intervals and p-values for exposure early graft function and outcome graft survival (HLA: human leukocyte antigen, DCD: donation after circulatory death, DBD: donation after brain death)

Supplementary Table 3:Coefficients, 95% confidence intervals and p-values for exposure early graft function and outcome death censored graft survival (BMI: body mass index, HLA: human leukocyte antigen, DCD: donation after circulatory death, DBD: donation after brain death)

| **Parameter** | **Co-efficient** | **Lower 95% CI** | **Upper 95% CI** | **p value** |
| --- | --- | --- | --- | --- |
| Donor Age | 0.01 | 0.01 | 0.01 | <0.001 |
| Donor hypertension | 0.06 | -0.02 | 0.14 | 0.15 |
| Graft number > 1 | 0.17 | 0.07 | 0.27 | <0.001 |
| Years on dialysis | 0.04 | 0.02 | 0.05 | <0.001 |
| Ischaemic time | 0.01 | 0.00 | 0.01 | 0.05 |
| Panel Reactive Antibodies | 0.00 | 0.00 | 0.00 | <0.001 |
| Recipient age (fractional polynomial) | -0.98 | -1.13 | -0.83 | <0.001 |
| Recipient age (fractional polynomial) | 0.11 | 0.09 | 0.13 | <0.001 |
| Primary renal disease |  |  |  |  |
| Glomerular Disease | 0.26 | 0.10 | 0.42 | <0.001 |
| Reflux | 0.24 | 0.04 | 0.44 | 0.02 |
| Hypertension | 0.24 | -0.01 | 0.48 | 0.06 |
| Diabetes | 0.35 | 0.09 | 0.62 | 0.01 |
| Other | 0.31 | 0.13 | 0.50 | <0.001 |
| Recipient BMI | 0.01 | 0.01 | 0.02 | <0.001 |
| Recipient Smoker | 0.31 | 0.22 | 0.40 | <0.001 |
| Recipient Peripheral vascular disease | 0.21 | 0.05 | 0.37 | 0.01 |
| Recipient Diabetes mellitus | 0.27 | 0.07 | 0.46 | 0.01 |
| 1 HLA-A mismatch | 0.15 | 0.03 | 0.27 | 0.01 |
| 2 HLA-A mismatches | 0.20 | 0.07 | 0.33 | <0.001 |
| 1 HLA-DR mismatch | 0.24 | 0.13 | 0.35 | <0.001 |
| 2 HLA-DR mismatches | 0.29 | 0.17 | 0.42 | <0.001 |
| Era of transplantation | -0.20 | -0.41 | -0.00 | 0.05 |
| Interaction terms |  |  |  |  |
| DCD Slow graft function | 0.20 | -0.30 | 0.71 | 0.43 |
| DCD Delayed Graft function | 0.59 | 0.17 | 1.01 | 0.01 |
| DBD Slow graft function | 0.29 | 0.14 | 0.44 | <0.001 |
| DBD Delayed Graft function | 0.40 | 0.27 | 0.53 | <0.001 |
| Living Slow graft function | 0.42 | 0.10 | 0.75 | 0.01 |
| Living Delayed Graft function | 0.66 | 0.24 | 1.08 | <0.001 |

| **Parameter** | **Co-efficient** | **Lower 95% CI** | **Upper 95% CI** | **p value** |
| --- | --- | --- | --- | --- |
| Donor Age | 0.01 | 0.00 | 0.01 | <0.001 |
| Donor hypertension | 0.09 | -0.01 | 0.18 | 0.07 |
| Donor Smoker | 0.08 | 0.01 | 0.16 | 0.03 |
| Graft number > 1 | 0.16 | 0.03 | 0.29 | 0.02 |
| Years on dialysis | 0.05 | 0.03 | 0.06 | <0.001 |
| Ischaemic time | 0.01 | 0.01 | 0.02 | <0.001 |
| Panel Reactive Antibodies | 0.00 | 0.00 | 0.00 | <0.001 |
| Recipient age | 0.05 | 0.05 | 0.05 | <0.001 |
| Recipient sex (male) | 0.02 | -0.06 | 0.1 | 0.61 |
| Primary renal disease |  |  |  |  |
| Glomerular Disease | -0.06 | -0.17 | 0.05 | 0.3 |
| Reflux | -0.1 | -0.28 | 0.08 | 0.28 |
| Hypertension | 0.16 | 0 | 0.33 | 0.06 |
| Diabetes | 0.24 | 0.06 | 0.42 | 0.01 |
| Other | 0.3 | 0.16 | 0.43 | <0.001 |
| Recipient Smoker | 0.3 | 0.22 | 0.37 | <0.001 |
| Recipient Peripheral vascular disease | 0.24 | 0.13 | 0.35 | <0.001 |
| Recipient Diabetes mellitus | 0.41 | 0.27 | 0.55 | <0.001 |
| Recipient Ischaemic heart disease | 0.25 | 0.16 | 0.34 | <0.001 |
| Recipient Cerebrovascular disease | 0.23 | 0.1 | 0.36 | <0.001 |
| Recipient Chronic liver disease | 0.27 | 0.15 | 0.39 | <0.001 |
| 1 HLA-DR mismatch | 0.05 | -0.04 | 0.14 | 0.25 |
| 2 HLA-DR mismatches | 0.11 | 0.01 | 0.21 | 0.02 |
| Era of transplantation | -0.25 | -0.42 | -0.07 | 0.01 |
| Interaction terms |  |  |  |  |
| DCD Slow graft function | 0.03 | -0.37 | 0.43 | 0.89 |
| DCD Delayed Graft function | 0.39 | 0.06 | 0.72 | 0.02 |
| DBD Slow graft function | 0.02 | -0.10 | 0.15 | 0.70 |
| DBD Delayed Graft function | 0.26 | 0.15 | 0.36 | <0.001 |
| Living Slow graft function | 0.44 | 0.12 | 0.75 | 0.01 |
| Living Delayed Graft function | 0.70 | 0.32 | 1.08 | <0.001 |

Patient survival by Early Graft Function

Supplementary Table 4:Coefficients, 95% confidence intervals and p-values for exposure early graft function and outcome patient survival (HLA: human leukocyte antigen, DCD: donation after circulatory death, DBD: donation after brain death)

| **Parameter** | **Co-efficient** | **Lower 95% CI** | **Upper 95% CI** | **p value** |
| --- | --- | --- | --- | --- |
| Donor age | -0.49 | -0.51 | -0.46 | <0.001 |
| Donor sex (male) | 3.41 | 2.77 | 4.04 | <0.001 |
| Donor BMI | 0.12 | 0.06 | 0.18 | <0.001 |
| Donor hypertension | -2.98 | -3.83 | -2.13 | <0.001 |
| Donor Smoker | 1.55 | 0.9 | 2.19 | <0.001 |
| Graft number > 1 | 1.18 | 0 | 2.36 | 0.05 |
| Years on dialysis | -0.39 | -0.52 | -0.26 | <0.001 |
| Ischaemic time | -0.17 | -0.25 | -0.09 | <0.001 |
| Panel Reactive Antibodies | -0.03 | -0.04 | -0.01 | <0.001 |
| Recipient age | -0.1 | -0.12 | -0.07 | <0.001 |
| Primary renal disease |  |  |  |  |
| Glomerular Disease | -0.81 | -1.75 | 0.13 | 0.09 |
| Reflux | -0.29 | -1.67 | 1.1 | 0.69 |
| Hypertension | 2.07 | 0.6 | 3.54 | 0.01 |
| Diabetes | -1.69 | -3.44 | 0.05 | 0.06 |
| Other | -0.47 | -1.63 | 0.7 | 0.43 |
| Recipient BMI | -0.48 | -0.55 | -0.42 | <0.001 |
| Recipient Smoker | -1.98 | -2.61 | -1.35 | <0.001 |
| Recipient Diabetes mellitus | 2.79 | 1.45 | 4.14 | <0.001 |
| Recipient Ischaemic heart disease | 0.65 | -0.23 | 1.52 | 0.15 |
| Recipient Cerebrovascular disease | 1.43 | 0.07 | 2.8 | 0.04 |
| Recipient Chronic liver disease | 1.02 | -0.15 | 2.19 | 0.09 |
| 1 HLA-DR mismatch | 0.05 | -0.04 | 0.14 | 0.25 |
| 2 HLA-DR mismatches | 0.11 | 0.01 | 0.21 | 0.02 |
| Era of transplantation | -0.25 | -0.42 | -0.07 | 0.01 |
| Interaction terms |  |  |  |  |
| DCD Slow graft function | -3.13 | -5.77 | -0.50 | 0.02 |
| DCD Delayed Graft function | -6.26 | -8.49 | -4.03 | <0.001 |
| DBD Slow graft function | -4.58 | -5.76 | -3.41 | <0.001 |
| DBD Delayed Graft function | -6.14 | -7.22 | -5.05 | <0.001 |
| Living Slow graft function | -5.18 | -7.78 | -2.58 | <0.001 |
| Living Delayed Graft function | -10.06 | -13.79 | -6.33 | <0.001 |

Estimated glomerular filtration rate by early graft function

Supplementary Table 5:Coefficients, 95% confidence intervals and p-values for exposure early graft function and outcome estimated glomerular filtration rate at 12 months (BMI: body mass index, HLA: human leukocyte antigen, DCD: donation after circulatory death, DBD: donation after brain death)

| **Parameter** | **Co-efficient** | **Lower 95% CI** | **Upper 95% CI** | **p value** |
| --- | --- | --- | --- | --- |
| Donor age | 0.01 | 0.01 | 0.01 | 0 |
| Donor sex (male) | -0.09 | -0.18 | 0 | 0.05 |
| Donor BMI | -0.01 | -0.02 | 0 | 0.03 |
| Graft number > 1 | 0.24 | 0.08 | 0.4 | 0 |
| Years on dialysis | 0.02 | 0 | 0.04 | 0.04 |
| Panel Reactive Antibodies | 0.01 | 0 | 0.01 | 0 |
| Recipient age | -0.02 | -0.02 | -0.01 | 0 |
| Recipient sex (male) | 0.12 | 0.03 | 0.22 | 0.01 |
| Recipient BMI | 0.03 | 0.02 | 0.04 | 0 |
| Recipient Smoker | 0.16 | 0.07 | 0.25 | 0 |
| 1 HLA-B mismatch | 0.11 | -0.03 | 0.26 | 0.13 |
| 2 HLA-B mismatches | 0.2 | 0.05 | 0.36 | 0.01 |
| 1 HLA-DR mismatch | 0.61 | 0.48 | 0.73 | 0 |
| 2 HLA-DR mismatches | 0.78 | 0.64 | 0.92 | 0 |
| Induction immunosuppression | -0.49 | -0.6 | -0.37 | 0 |
| Interaction terms |  |  |  |  |
| DCD Slow graft function | 0.28 | -0.02 | 0.59 | 0.07 |
| DCD Delayed Graft function | 0.41 | 0.18 | 0.63 | <0.001 |
| DBD Slow graft function | 0.25 | 0.08 | 0.42 | <0.001 |
| DBD Delayed Graft function | 0.56 | 0.41 | 0.7 | <0.001 |
| Living Slow graft function | 0.12 | -0.23 | 0.47 | 0.5 |
| Living Delayed Graft function | 0.77 | 0.33 | 1.2 | <0.001 |
|  |  |  |  |  |
|  |  |  |  |  |

Supplementary Table 6: Coefficients, 95% confidence intervals and p-values for exposure early graft function and outcome rejection episodes at 12 months (BMI: body mass index, HLA: human leukocyte antigen, DCD: donation after circulatory death, DBD: donation after brain death)
